# Knowledge of vaccine handlers and status of cold chain and vaccine management in primary health care facilities of Tigray region, Northern Ethiopia: Institutional based cross-sectional study

**DOI:** 10.1371/journal.pone.0269183

**Published:** 2022-06-01

**Authors:** Hailay Gebretnsae, Tsegay Hadgu, Brhane Ayele, Equbay Gebre-egziabher, Mulugeta Woldu, Mulugeta Tilahun, Alemnesh Abraha, Tewolde Wubayehu, Araya Abrha Medhanyie

**Affiliations:** 1 Tigray Health Research Institute, Mekelle, Tigray, Ethiopia; 2 College of Health Sciences, School of Public Health, Mekelle University, Mekelle, Tigray, Ethiopia; University of Washington, UNITED STATES

## Abstract

**Background:**

Ethiopia federal ministry of health has been working on increasing access to immunization service by deploying solar refrigerators to primary health care facilities. However, there is limited evidence on cold chain and vaccine management status. Therefore, the objective of this study was to assess knowledge of vaccine handlers and status of cold chain and vaccine management and their associated factors in primary health care facilities of Tigray region Northern Ethiopia.

**Methods:**

Institutional based cross-sectional study was conducted in four randomly selected districts of Tigray region, Northern Ethiopia. In each selected district, all primary health care facilities with functional vaccine refrigerators were included in the study. Data were collected using a pre-tested semi-structured questionnaire. The collected data were entered into Epi-data version 3.1 and then exported to Statistical Package for Social Sciences (SPSS) version 21 for analysis. All variables with p-value of < 0.25 in bivariate logistic regression analysis were included in multi-variable model to identify predictors of the dependent variables.

**Results:**

In this study, fifty Primary Health Care Facilities (PHCFs) were included with a response rate of 94.4%. The overall level of good knowledge of vaccine handlers and good status of cold chain and vaccine management were 48% (95% CI; 30.7%-62%) and 46% (95%CI; 26.1%-61.3%) respectively. Receiving training on cold chain and vaccine management (AOR = 5.18; 95%CI: 1.48–18.18) was significantly associated with knowledge of vaccine handlers. Furthermore, receiving supportive supervision (AOR = 4.58; 95%CI: 1.04–20.17) and good knowledge of vaccine handlers (AOR = 10.97; 95%CI: 2.67–45.07) were significant associated with cold chain and vaccine management.

**Conclusions:**

This study showed that knowledge of vaccine handlers on cold chain and vaccine management was poor. Similarly, the cold chain and vaccine management status was also poor. Therefore, on-site training should be provided to vaccine handlers to increase their knowledge, so as to improve their practices on cold chain and vaccine management. In addition, Programme based supportive supervision is needed to improve cold chain and vaccine management.

## Introduction

Vaccines are biological products that are sensitive to heat, light and freezing, which requires them to be stored within recommended temperature ranges in a cold chain system as their potency cannot be restored if they have been exposed to heat and cold temperature [1, 2]. Cold chain is a system used for storing vaccines in good condition [2]. It consists of a series of storage and transport links, all designed to keep vaccines within an acceptable temperature range until it reaches the users [3]. Wherever vaccines are stored, a system of vaccines management must be in place to record vaccines received, dispatched, used and wasted [4]. This will make sure that vaccines are used before their expiry date and reach Vaccine Vial Monitor (VVM) discarding stage [5]. If all vaccines are properly recorded at receipt and issue, vaccines under-stock or over-stock won’t happen [4].

Proper cold chain management system is the backbone for success of an Expanded Programme on Immunization (EPI), and thus there is a crucial need to preserve the potency and safety of the vaccine to ensure reduction in childhood deaths due to Vaccine Preventable Diseases (VPDs) [3, 6–8]. Vaccines must be arranged inside cold chain equipment in good condition with minimum risk of exposure to damaging temperatures [2]. Improper storing and handling of vaccines can reduce vaccine potency, resulting in inadequate immune responses and poor protection against VPDs [3].

Cold Chain and Vaccine Management (CCVM) remains an exceptionally challenging component of EPI [3, 6, 9]. These challenges are highly faced especially in lower levels of the health system for different reasons like: problems related to vaccine storage, lack of temperature measurement devices, lack of continuous temperature documentation, inadequate refrigerators, impropriate storage practices, lack of designated personnel, and insufficient staff training [10]. Additionally, knowledge gap of vaccine handlers on vaccine wastage control, stock management, distribution planning and monitoring are among the gaps on vaccine management [5, 11, 12].

Several factors are associated with cold chain and vaccine management. These include receiving training, supportive supervision, and level of education among health care providers [13, 14]. Furthermore, inadequate knowledge is common among health care professionals, especially in remote areas [9]. Training health care providers significantly improve cold chain monitoring [10, 15], especially in-service training [9, 16]. A well-trained vaccine handler and familiar with cold chain and vaccine management are critical to ensure the potency of vaccine and safety [3].

Ethiopian ministry of health has been working on different measures to improve access and coverage of immunization services by deploying solar refrigerators to primary health care facilities. However, there is limited evidence on cold chain and vaccine management status and still VPDs are being observed as outbreaks in Tigray region. Therefore, this study aimed to assess the knowledge of vaccine handlers and status of CCVM and their associated factors at primary health care facilities in selected districts of Tigray region, Northern Ethiopia. The findings of the study will help to generate evidence based information to health care providers and program managers to improve cold chain and vaccine management system in the region.

## Methods

### Study setting and period

The study was conducted in four districts (Tahtay-Maychew, Werie-Leke, Saharti-Samre and Raya-Alamata) of Tigray region, Northern Ethiopia from May to June, 2019. Tigray region is one of the nine regional states of Ethiopia found in the north. The region has seven zones which are further divided into 52 districts. According to the 2007 census, the estimated population was 107,284 in Tahtay-Maychew, 175,197 in Werie-Leke, 146,700 in Saharti-Samre and 99,635 in Raya-Alamata in 2019. In the selected districts, there are 73 health posts, 19 health centers and 3 primary hospitals that provide health services to the community (S1 Table). The Ethiopian health service is structured in three tier system; namely primary, secondary and tertiary level health care. The primary level health care includes primary hospital, health center and health post; secondary level health care refers to general hospital and tertiary level health care refers to specialized hospital. Vaccination is provided at all level of health system mainly in the primary level health care. Vaccine supply chain in Tigray region indicates that regional store is supplied by central/national store. The regional store supplies to district stores and the district store supplies to health facilities where vaccination takes place.

### Study design

Institution based cross-sectional study was conducted to assess knowledge of vaccine handlers and cold chain and vaccine management status in primary health care facilities of Tigray region, Northern Ethiopia.

### Study participant

One vaccine handler per Primary Health Care Facility (PHCF) (responsible person for managing the cold chain and vaccine from the selected health posts, health centers and primary hospitals) was included in the study. In case of more than one eligible vaccine handlers, one study participant was selected randomly using lottery method.

### Sampling methods and procedure

From the total of 52 districts in Tigray region, 18 districts were labeled VPDs outbreak prone districts based on regional surveillance report and frequent breakage of the cold chain. From the labeled 18 VPDs outbreak prone districts, four districts were selected by simple random sampling using lottery method. In each selected district, all (50) PHCFs (28 health posts, 19 health centers and 3 primary hospitals) with functional vaccine refrigerator were included in the study. Three health posts were not included in the study due to they were closed at the time of data collection (S1 Fig).

### Data collection procedures and data quality assurances

A semi-structured questionnaire was adapted from World Health Organization (WHO) assessment tools and related studies [13, 17, 18]. The questionnaire had six different parts: 1) socio-demographic characteristics 2) knowledge of vaccine handlers 3) cold chain status 4) vaccine management 5) receiving supportive supervision 6) availability of vaccine equipment, recording and reporting tools (S1 File). The data were collected through face-to-face interview with vaccine handlers. Furthermore, direct observation and document review were conducted to check cold chain and vaccine status, availability and functionality of cold chain equipment, and availability of recording and reporting tools. Eight EPI focal persons who have experience in data collection and trained on CCVM were recruited from non-study districts for the data collection. Training was given for two days to the supervisors and data collectors on how to facilitate the data collection. Pre-test was conducted in 5% of primary health care facilities in a district other than the study sites. Daily data quality-check was done during the data collection period by two supervisors and two primary investigators.

### Measurement of variables

#### Knowledge of vaccine handlers

This dependent variable was computed from 16 knowledge related questions on cold chain and vaccine management (know the recommended storage temperature range of vaccines (between +2°C to +8°C) in the PHCF, know the recommended storage temperature range of diluents, know how to pack ice pack in vaccine carrier, know how to prevent vaccine freezing during transport, know most heat sensitive vaccines, know most light sensitive vaccines, know most cold sensitive vaccines, correctly read and interpret VVM stages, correctly demonstrate and interpret shake test, know vaccines eligible for four weeks open vial policy, know Early Expiry First Out (EEFO) principle based on VVM status, know EEFO principle based on expiry date, know how to organize old and new vaccines, know vaccines should be stored only for a maximum of one month in PHCF, know diluents and vaccine should be from same manufacturer, and know how to calculate the vaccines wastage rate). Those who scored greater than the mean were considered as having good knowledge and those who scored below the mean were considered as having poor knowledge with value of one and zero respectively (S2 Table).

#### Cold chain and vaccine management status

This dependent variable was obtained from 13 indicators on cold chain and vaccine management (proper arrangement of vaccines, proper packing of diluents, proper packing of ice packs, do not keep expired vaccines in the refrigerators, do not keep vaccines with VVM that reached discarding stage, recording temperature twice daily, use foam pad always during immunization sessions, check physical stock every month before ordering the next request vaccines, use standard vaccine requisition format, proper registration of all vaccines in the stock register, did not experience under-stock of any vaccine within the last six months, did not experience over-stock of any vaccine within the last six months, and calculate vaccine wastage rate every month). Those who scored greater than the mean were considered as having good cold chain and vaccine management status and those who scored below the mean were considered as having poor cold chain and vaccine management status with value of one and zero respectively (S3 Table).

#### Availability of necessary cold chain equipment

This is a dichotomous variable with a value of “1 = Yes”, if the Primary Health Care Facilities (PHCFs) had temperature monitoring device, foam pad, at least two vaccine carriers and 8 ice packs and “0 = No”, if at least one of the above mentioned equipment was missed.

#### Availability of necessary recording and reporting tools

This was a dichotomous variable with a value of “1 = Yes”, if the PHCFs had temperature recording charts, vaccine stock register and standard vaccine requisition format and “0 = No” if at least one of the above mentioned tools was missed.

### Data management and analysis

Data were entered in to Epi-data version 3.1 and then exported to Statistical Package for Social Sciences (SPSS) version 21 for analysis. Data cleaning and checking were done before analysis. Knowledge of vaccine handlers and cold chain and vaccines management status were computed separately and the total scores were recorded as dichotomous variable (good vs. poor) based on their mean scores as stated in the measurement of variables. Frequency distribution and percentages were presented using frequency tables. Logistic regression analysis was done to see the association of independent variables with the dependent variables (knowledge of vaccine handlers and cold chain and vaccines management status). All variables with a p-value of < 0.25 in the bivariate analysis were fitted in to the multi-variable logistic regression model to identify predictor variables. Finally the variables with p-value less than 0.05 with 95% confidence interval were identified as determinant factors.

### Ethical consideration

The study protocol was reviewed and approved by institutional ethical review board of Tigray Health Research Institute (Reference number: THRI RM/150-05/2019). Letter of support was obtained from Tigray Regional Health Bureau (TRHB) and the selected district health offices. Respondents involved in the study were fully informed about the objectives and purpose of the study, and written consent was obtained from each participant before the interview. Participation in the interview was on voluntary basis and respondents were informed that they have the right to withdraw from interview at any stage of the interview. Personal identifiers were not used in the questionnaire and confidentiality of the information was assured.

## Results

### Characteristics of PHCFs and vaccine handlers

A total of 53 primary health care facilities were eligible and 50 (94.4%) of them were included in the study. Among these, 28 (56%) were health posts. The mean age of the respondents was 29.48 (±7.09 Standard Deviation (SD) years, above three-fourth (76%) of the respondents were diploma holders and 35(70%) of them were currently in union in their marital status (Table 1).

**Table 1:** Socio-demographic characteristics of PHCFs and respondents in primary health care facilities of Tigray region, Northern Ethiopia, 2019 (N = 50).

Variable	Number	Percent
**Types of primary health care facility**		
Health posts	28	56
Health centers	19	38
Primary hospitals	3	6
**Mean age**	29.48(±7.09 SD) years	
**Sex**		
Male	8	16
Female	42	84
**Educational status**		
Certificate /Level III	7	14
Diploma/Level IV	38	76
Bachelor degree	5	10
**Marital status**		
Currently not in union *	15	30
Currently in union	35	70
**Type of profession**		
Health Extension Worker(HEW)	27	54
Nurse	19	38
Other **	4	8
**Mean of total work experience**	7.72(± 6.05 SD) years	
**Work experience related to cold chain management (Median ± (Inter Quartile Range (IQR))**	1.5 (±3 IQR) years	
**Received training on cold chain and vaccine management**		
Yes	30	60
No	20	40
**Received supportive supervision on cold chain and vaccine management in the last 6 months**		
Yes	18	36
No	32	64
**Having vaccines micro plan**		
Yes	6	12
No	44	88
**Having proper vaccines supply chain system**		
Yes	5	10
No	45	90
**Conducted cold chain equipment inventory**		
Yes	7	14
No	43	86

*(single, widowed and separated)

** (midwife, health officer and pharmacist)

### Knowledge of vaccine handlers on cold chain and vaccine management

Above half, 26 (52%) of vaccine handlers did not demonstrate correctly how to pack ice pack to vaccine carrier. Almost two-third 32(64%) of respondents knew correct interpretation of VVM stages. However, only 6(12%) of participants knew how to perform the shake test and the reason for the application of the shake test.

Regarding knowledge on vaccine management, 26 (52%) and 33 (66%) of respondents knew Early Expiry First Out (EEFO) principle based on VVM stage and expiry date status respectively. However, only 11(22%) of vaccine handlers knew how to calculate the wastage. Overall, the mean and Standard Deviation (SD) of knowledge score was 7.52±3.16 and only 48% (95% CI; 30.7%-62%) of the vaccine handlers had good knowledge on cold chain and vaccine management (Table 2).

**Table 2:** Knowledge of vaccine handlers on cold chain and vaccine management in primary health care facilities of Tigray region, Northern Ethiopia, 2019 (N = 50).

Indicator		Number	Percent
knew the recommended storage temperature range of vaccines (between +2°C to +8°C) in PHCF		40	80
Knew the recommended storage temperature range of diluents (cooled to 2–8°C)		13	26
Correctly demonstrated how to pack ice pack in vaccine carrier		24	48
Knew how to prevent vaccine freezing during transport		28	56
Named most heat sensitive vaccines (OPV, Measles and BCG)		27	54
Named most cold sensitive vaccines (DPT-HepB-Hib, PCV, TT, IPV, and Rota)		12	24
Named most light sensitive vaccines (BCG and measles)		13	26
Correctly interpreted VVM stages		32	64
Correctly demonstrated and interpreted shake test		6	12
knew vaccines eligible for four weeks open vial policy(TT, IPV and OPV)		23	46
Knew Early Expiry First Out (EEFO) principle based on VVM status		26	52
Knew Early Expiry First Out (EEFO) principle based on expiry date		33	66
Knew how to organize old and new vaccines to facilitate use of older vaccines first		23	46
Knew vaccines should be stored only for a maximum of 1 month in PHCF		45	90
Knew diluents and vaccine should be from same manufacturer		20	40
Knew how to calculate vaccines wastage rate		11	22
Mean score of knowledge on cold chain and vaccine management		7.52 (±3.16 SD)	
Overall knowledge on cold chain and vaccine management	Good	24	48
	Poor	26	52

### Status of cold chain in PHCFs

Primary Health Care Facilities (PHCFs) had improper storage of: vaccines 43(86%), diluents 30(60%) and ice packs 23(46%). Vaccines were stored within refrigerators with non-vaccine products (like laboratory reagents, drugs, and other drinks and foods) (30%; n = 15), with expired vaccines (16%; n = 8) and with VVM that reached discarding stage (34%; n = 17). About 60% (n = 30) of the PHCFs were found opened multi-dose vials within the refrigerators and one-third 36.7% (n = 11) of them were placed without clearly labeled.

During the period of three months following the study period; daily temperature was recorded in 17(34%) of the PHCFs. Among those, below three-fourth(23.5%) of them had completed and up-to-dated temperature recording, and above two-third (70.6%) of the PHCFs recorded at least one abnormal temperature (58.8% of them exposed to temperature higher than 8°C and 41.2% of them exposed to cool temperature lower than +2°C). However, only 11.8% of them had formally reviewed the temperature records and took remedial actions. Furthermore, out of the total fifty PHCFs, only 15(30%) had used standard vaccine requisition format for ordering and receiving vaccines, and 26% (n = 13) and 28% (n = 14) of them experienced under stock and over-stock for at least one vaccine within the last six months respectively. In general, the mean and SD of cold chain and vaccine management status was 5.60 ± 2.21 and only 46% (95% CI; 26.1%-61.3%) of the PHCFs had good cold chain and vaccine management status (Table 3).

**Table 3:** Cold chain and vaccine management status in primary health care facilities of Tigray region, Northern Ethiopia, 2019, (N = 50).

Indicator		Number	%
Vaccines properly arranged inside the refrigerators		7	14
Diluents properly packed inside the refrigerators		20	40
Ice packs properly packed inside the refrigerators		27	54
PHCFs did not keep expired vaccines in refrigerators		42	84
PHCFs did not keep vaccines with VVM that reached discarding stage		33	66
Record temperature twice daily		17	34
Use foam pad always during immunization sessions		13	26
Check physical stock every month before ordering the next request vaccines		21	42
Use standard vaccine requisition format for ordering and receiving vaccines		15	30
All vaccines properly registered in the stock register		6	12
PHCFs did not experience under- stock of any vaccine within the last six months		37	70
PHCFs did not experience over-stock of any vaccine within the last six months		36	72
Calculate vaccine wastage rate every month within the last six months		6	12
Mean score of cold chain and vaccine management status		5.60 (± 2.21 SD)	
Overall cold chain and vaccine management status	Good	23	46
	Poor	27	54

### Availability of infrastructure and cold chain equipment

Of the total, 21(42%) of the PHCFs had access to electricity power supply. Twenty-seven (54%) of the PHCFs had functional temperature monitoring device. Only 48% (n = 24), 32% (n = 16), and 28% (n = 14) of the PHCFs had vaccine stock register, vaccine requisition format and temperature recording chart respectively (Table 4).

**Table 4:** Availability of infrastructure and cold chain equipment in primary health care facilities of Tigray region, Northern Ethiopia, 2019 (N = 50).

Variable	Number	Percent
**Type of power supply for vaccine refrigerators**		
Electricity	21	42
solar	29	58
**Total numbers of functional refrigerators in the PHCF**		
1	28	56
2–3	18	36
≥ 4	4	8
**HF with at least one dysfunctional refrigerator**		
Yes	25	50
No	25	50
**Availability of functional temperature monitoring device**		
Yes	27	54
No	23	46
**Availability of ice parks in the PHCF**		
≥ 8	40	80
< 8	10	20
**Numbers of vaccine carrier in the PHCF**		
None	4	8
1–2	18	36
≥ 3	28	56
**Availability of foam pad**		
Yes	17	34
No	33	66
**Availability of necessary cold chain equipment**		
Yes	4	8
No	46	92
**Availability of vaccine stock register**		
Yes	24	48
No	26	52
**Availability of standard vaccine requisition format**		
Yes	16	32
No	34	68
**Availability of temperature recording chart**		
Yes	14	28
No	36	72
**Availability of necessary recording and reporting tools**		
Yes	10	20
No	40	80
**Cold chain equipment inventory checklist**		
Yes	4	8
No	46	92
**Presence of the national guidelines on immunization**		
Yes	17	34
No	33	66
**Presence of written instructions on the VVM reading**		
Yes	16	32
No	34	68

### Factors associated with knowledge of vaccine handlers

Four variables with a p-value < 0.25 in bivariate analysis were entered into the multi-variable logistic regression model to identify factors associated with knowledge of vaccine handlers. Finally, receiving training on cold chain and vaccine management was the only significantly predictor variable with knowledge of vaccine handlers. The odds of having good knowledge among vaccine handlers who had received training on cold chain and vaccine management was five times (AOR = 5.18; 95%CI: 1.48–18.18) higher than their counterparts (Table 5).

**Table 5:** Logistic regression analysis of selected variables with knowledge of study participants on cold chain and vaccine management in primary health care facilities of Tigray region, Northern Ethiopia, 2019(N = 50).

Variables	Level of knowledge		COR (95% CI)	AOR) (95% CI)
	Good (%)	Poor (%)		
**Age**				
**≤** 29 years	17(54.8)	14(45.2)	2.08(0.65–6.71)	1.98(0.54–7.25)
> 29 years	7(36.8)	12(45.2)	1	1
**Work experience related to cold chain management**				
< 1year	7(36.8)	12(63.2)	2.10(0.65–6.71)	1.92(0.48–7.76)
≥ 1year	17(54.8)	14(45.2)	1	1
**Received training on cold chain and vaccine management**				
Yes	19(63.3)	11(36.7)	**5.18(1.48–18.18)** *	**5.18(1.48–18.18)** *
No	5(25.0)	15(75.0)	1	1
**Received supportive supervision on cold chain and vaccine management**				
Yes	12(66.7)	6(33.3)	3.33(0.99–11.22)	1.77(0.39–8.10)
No	12(37.5)	20(62.5)	1	1
**Presence of national EPI guideline**				
Yes	12(70.6)	5(29.4)	**4.20(1.19–14.83)** *	2.93(0.77–11.20)
No	12(36.4)	21(63.4)	1	1

*statistically significant at 0.05<p<0.01,

** statistically significant at 0.01< p<0.001,

*** statistically significant at p<0.001

### Factors associated with cold chain and vaccine management

In a multi-variable logistic regression analysis, receiving supportive supervision and vaccine handlers who had good knowledge were significantly associated with good status of cold chain and vaccine management after adjusting for potential confounding variables. Vaccine handlers who had received supportive supervision in the last 6 months were 4.58 times more likely to have good cold chain and vaccine management practice than those vaccine handlers had not received supportive supervision in the last 6 months (AOR = 4.58, 95%CI: 1.04–20.17). The odds of having good cold chain and vaccine management practices among vaccine handlers who had good knowledge were almost 11 times higher than those vaccine handlers who had poor knowledge (AOR = 10.97, 95%CI: 2.67–45.07) (Table 6).

**Table 6:** Logistic regression analysis of selected variables with cold chain and vaccine management in primary health care facilities of Tigray region, Northern Ethiopia, 2019(N = 50).

Variables	Cold chain and vaccine management status		COR (95% CI)	AOR) (95% CI)
	Good (%)	Poor (%)		
**Marital status**				
Currently not in union *	9(60.0)	6(40.0)	2.25(0.66–7.73)	0.81(0.14–4.70)
Currently in union	14(40.0)	21(60.0)	1	1
**Received training on cold chain and vaccine management**				
Yes	18(60.0)	12(40.0)	**4.50(1.29–15.68)** *	1.69(0.36–7.89)
No	5(25.0)	15(75.0)	1	1
**Received supportive supervision on cold chain and vaccine management**				
Yes	5(27.8)	13(72.2)	**5.72(1.60–20.45)** **	**4.58(1.04–20.17)** *
No	22(68.8)	10(31.3)	1	1
**Knowledge on cold chain and vaccine management**				
Good	18(75.0)	6(25.0)	**12.60(3.29–48.29)** ***	**10.97(2.67–45.07)** **
Poor	5(19.2)	21(80.8)	1	1

*statistically significant at 0.05<p<0.01,

** statistically significant at 0.01< p<0.001,

*** statistically significant at p<0.001

## Discussion

In this study, 80% of vaccine handlers knew the recommended range of temperature for vaccine storage. This was comparable with a study done in central Ethiopia (78.4%) [19]. However, our finding was lower when compared with studies conducted in Oromia, Ethiopia (96.9%) [20] and Malaysia (95.5%) [21]. On the hand, our finding was higher than in Bale and Gurage zone, Ethiopia (67.8% and 71.1%) [22, 23], Cameroon (68.5%) [13], Mozambique (52%) [24] and Nigeria (52%) [25]. This difference may be due to the difference in educational level, profession type, training exposure and year of experience of the study participants.

In the present study, only 12% of respondents demonstrated and interpreted the shake-test correctly. Our finding was much lower than reported from previous similar studies in Ethiopia ranging from (36.2% to 53.3%) [20, 22, 26], India (40%) [27] and Nigeria (51.6%) [25]. Furthermore, two-third (64%) of respondents had interpreted correctly the vaccine vial monitor (VVM) stages. This was not very different from the proportion documented in Gojam zone, Ethiopia (58.3%) [26], but, it is higher than study done in Nigeria (45.3%) [25]. This gap could be due to lack of practical sessions and follow-up during trainings and supportive supervision.

In this study, the types of vaccines that were most sensitive to freeze, light and heat were correctly reported by 24%, 26% and 54% of the respondents, respectively. Our finding was lower when compared with another studies conducted in Oromia and Bale zone of Ethiopia [20, 22]. However, the knowledge on most sensitive to heat was slightly higher than in Oromia, Ethiopia (52.9%) [20]. Furthermore, regarding the knowledge about vaccines that are eligible to the four weeks open vial policy, 46% of vaccine handlers correctly listed three vaccines (TT, IPV and OPV). This finding is almost the same with a report from Cameroon (43.8%) [13]. However, our finding was lower when compared with finding reported from central Ethiopia and Oromia (62.9% and 64.6%) [19, 20].

Our study showed that the arrangement of vaccines in the compartment of the refrigerators was improper in 86% of the PHCFs. If the vaccine is not properly arranged, potency of vaccines will be lost completely, and it will be useless. This problem was higher than other study conducted in Bale zone of Ethiopia (60%) [22].

Ideally, refrigerators containing vaccines should not be used to store non-vaccine products, expired vaccines and vaccines with VVM that reached discarding stage. However, our study showed that vaccines were sharing space with none-vaccine products (laboratory reagents, drugs, and other drinks and foods) (30%), expired vaccines (16%) and vaccine with VVM that reached discarding stage (34%). This constraint had also been documented in Nigeria, which reported that expired vaccines (9.5%), vaccine with VVM that reached discarding stage (16.3%) and none-vaccine products (71.4%) were found with vaccines within the refrigerators [25]. None-vaccine products and vaccines with VVM that reached discarding stage stored with vaccines had also been reported by other related studies [15, 18, 22–26, 28]. Storing vaccines with none- vaccine products might expose the refrigerators for repeated opening which affect to maintain the recommended range of temperature and potency of the vaccines [25]. Furthermore, putting expired and vaccine with VVM that reached discarding stage with vaccines could also be liable to mistakenly administering such vaccines by health providers during immunization session.

In the current study, only 17(34%) of PHCFs had recorded daily temperature during the last 3 months following data collection; among which only 23.5% of them had proper recording practice (complete and up-to-date). This finding is lower than reported from Bale, Gojam and Gurage zone of Ethiopia (57.1%,58.3% and 85.7% respectively), Thailand (61.1%) and India (95%) of health facilities had recorded temperature twice daily [22, 23, 26, 29, 30]. However, our finding on proper temperature recording practice is higher than the reported in Bale, in which none of health facilities had complete and update temperature recording practice [22], but it is lower than the reported from Cameroon, in which 49% of health facilities had completed and up-to-dated temperature recording practice [18]. This difference may be due the difference on availability of temperature recording chart, training status and sampling variation. Furthermore, of the total 17 PHCFs which had recorded daily temperature, 70.6% of them had recorded at least one abnormal temperature range during the last 3 months of the data collection. This finding was almost the same with study done in Mozambique (69.2%) of the health facilities had outside the recommended temperature range [24]. However, it is higher than the study conducted in Central Ethiopia (27.3%) and Bale (17.14%) [19, 22] and Cameroon (26.9%) [18]. This difference may be due the difference in the total duration of sampling on reviewed temperature recording.

Having vaccines micro plan, proper vaccine supply chain system and conducting cold chain equipment inventory are critical for effective cold chain and vaccine management. However, in this study only 12%, 10% and 14% of the PHCFs had vaccines micro plan, proper vaccine supply chain system and conducted cold chain equipment inventory respectively. This could result in improper cold chain and vaccine management including vaccines under or over stocks. Our finding on having vaccines micro plan was lower than the reported from Cameroon (64.3%) of health facilities had vaccines micro plane [13]. However, the proper vaccines supply chain system is nearly the same with the reported from low and lower-middle-income countries, which revealed that the proper vaccine distribution down the supply chain was attained in only 15% [1]. Furthermore, PHCFs had experienced at least one vaccine under stock (26%) and over-stock (28%) in the last six months prior to this assessment. The problem of vaccine under stock was lower than the finding from South Africa, which was reported, (77%) of the health facilities had at least one vaccine stock out [31]. However, it is higher than the reported in Cameroon (11.9%) of the health facilities had run short of stock of vaccines [13]. This difference may be due the difference in the sampling methods and study settings.

This study showed that 48% of vaccine handlers had a good knowledge on cold chain and vaccine management. This figure is nearly consistent with previous related studies from Ethiopia, Gurage (51.3%), Oromia (53.5%), Bale (54.6%) and central Ethiopia (56%) [19, 20, 22, 23] and Nigeria (43%) [32]. However, our finding was lower when compared with an another study conducted in Malaysia (78.7%) [21]. On the other hand, this finding was higher than the finding from Gojan zone of Ethiopia (38.3% [26] and Nigeria (36%) [33]. The discrepancy may be due to a difference in operational definition, study settings and professional category of the study participants in which especially the Malaysian study was solely among general practitioners (GPs).

In this study, the good status of cold chain and vaccine management was 46%. This finding is almost consistent with a study done in Oromia, Ethiopia (48.8%) [20]. However, our finding is lower than the finding from Gojan, Ethiopia (58.3%) [26] and Nigeria (66.1% and 73.9%) [14, 32]. However, our finding is higher than the studies done in India (35%) [34] and Bahir Dar, Ethiopia (28.6%) [35]. This difference may be due to the difference in the selection of the indicators to measure the status of cold chain and vaccine management and the way of categorization of the variable.

In the current study, receiving training on cold chain and vaccine management was the only significantly associated factor with knowledge of vaccine handlers. This finding is supported with previous studies which revealed that vaccine handlers who had received training had better knowledge than vaccine handlers who had not received training [20, 23, 30, 33]. Training could increase the vaccine handlers’ curiosity and attention to apply the principles obtained from the training. This in-turn could increase the knowledge of vaccine handlers on cold chain and vaccine management.

The success of cold chain and vaccine management depends on the knowledge of cold chain handlers [27]. In this study, having good knowledge on cold chain and vaccine management was significantly associated with good cold chain and vaccine management status. The finding is consistent with previous studies [26, 32, 35]. This could be the possible explanation that if vaccine handlers have good knowledge, they may have good motivation, and feel responsibility and accountability to practice the proper cold chain and vaccine management.

In the current study, receiving supportive supervision was also another determinant factor for good cold chain and vaccine management status. This finding is supported with previous studies which revealed that supportive supervision was significant determinant of good practice of cold chain management [14]. Other interventional studies from India reported that cold chain and vaccine management practice was significantly improved after the implementation of supportive supervision program [27, 36]. This may be due to the fact that during supportive supervision, corrections of errors in cold chain and vaccine management are made on the spot wherever required, and vaccine handlers are encouraged and motivated to solve the feed backs given in to an action.

### Strength and limitation of the study

Strength of this study is the fact that data were collected through different data collection techniques like; interviews, observations and document review. Furthermore, it is less likely to be affected by bias as we did not inform the facilities ahead of the visit. However, due to this reason we may fail to get appropriate person who could have been waiting there in the health facilities, which could in-turn affect the result of the study, especially on the knowledge part. Another limitation is the sample size of the study was relatively small which may have affected both the precision of the proportion estimates as well as the statistical inferences of the modeling. Therefore, caution is needed during interpretation of these results.

## Conclusions

Our study showed that knowledge of vaccine handlers on cold chain and vaccine management was poor. Similarly, the cold chain and vaccine management status was also poor. Therefore, on-site training should be provided to vaccine handlers to increase their knowledge, so as to improve their cold chain and vaccine management practices. In addition, Programme based supportive supervision is needed to improve cold chain and vaccine management.

## Supporting information

S1 Fig S1 Fig which was used as schematic presentation of sampling procedure. (TIF)

S1 File This S1 File questionnaire which was used to collect a data. (DOCX)

S1 Table This S1 Table contains the availability of functional vaccine refrigerators in selected districts. (DOCX)

S2 Table This S2 Table contains the variables which were used to compute the knowledge of vaccine handlers. (DOCX)

S3 Table This S3 Table contains the variables which were used to compute the cold chain and vaccine management status. (DOCX)

S1 Checklist This S1 Checklist contains the STROBE check list of the manuscript. (DOCX)
