# Supplementary figures and images for: Knowledge of vaccine handlers and status of cold chain and vaccine management in primary health care facilities of Tigray region, Northern Ethiopia: Institutional based cross-sectional study
Source: PLoS One. 2022 Jun 1;17(6):e0269183. doi: 10.1371/journal.pone.0269183 (PMC9159613; doi:10.1371/journal.pone.0269183)

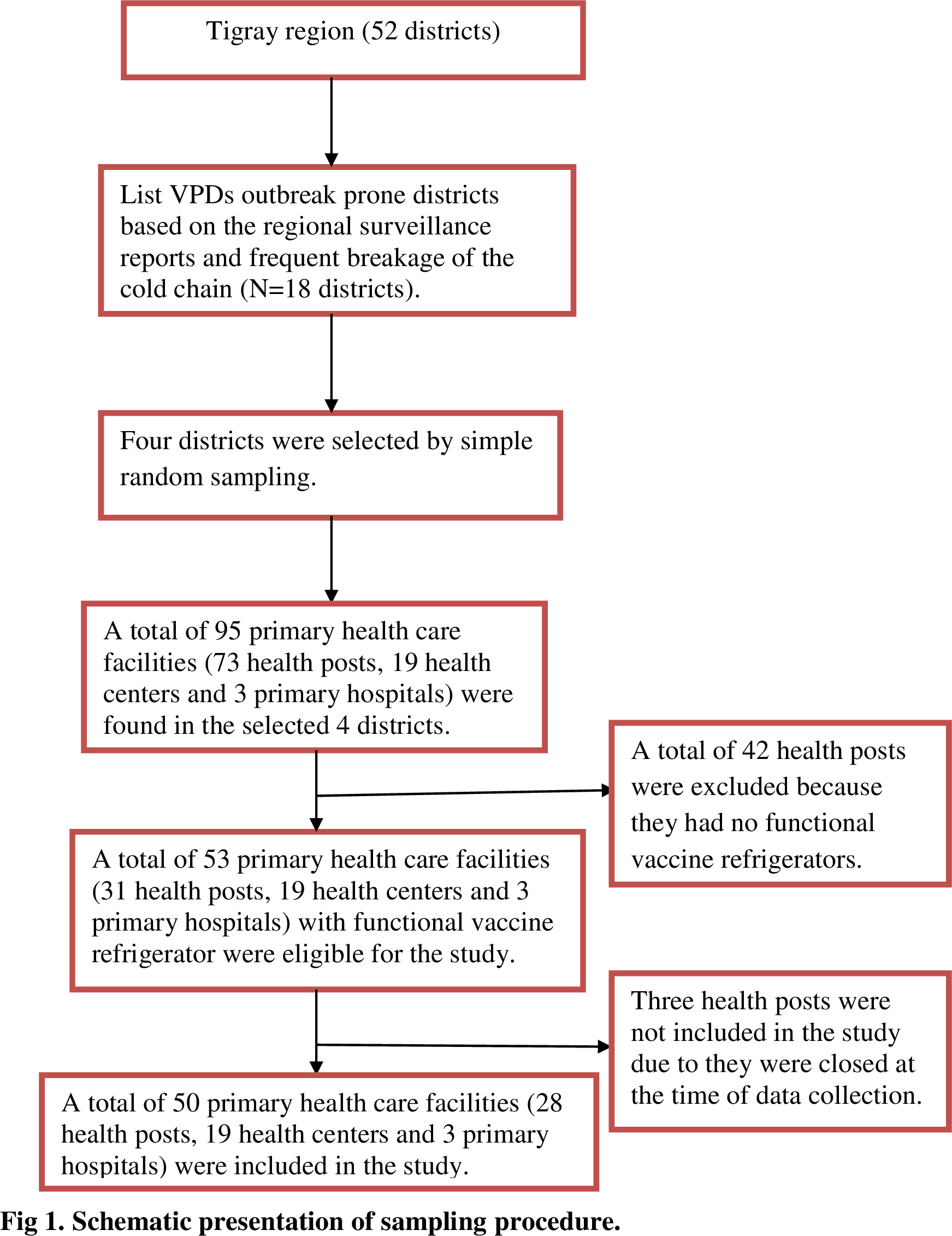

Supplement: S1 Fig — (TIF) [file pone.0269183.s001.tif]
