# Supplementary material for: Knowledge of vaccine handlers and status of cold chain and vaccine management in primary health care facilities of Tigray region, Northern Ethiopia: Institutional based cross-sectional study
Source: PLoS One. 2022 Jun 1;17(6):e0269183. doi: 10.1371/journal.pone.0269183 (PMC9159613; doi:10.1371/journal.pone.0269183)
Supplement: S1 File — (DOCX) [file pone.0269183.s002.docx]

**Tigray Health Research Institute**

**I Information sheet and consent form**

**Assessment on knowledge of vaccine handlers and status of cold chain and vaccine management in primary health care facilities of Tigray region, Northern Ethiopia**

Name of District ………………………..Name of health facility…………………………….

Questionnaire identification number/code...............................

**Information sheet**

Hello! My name is ______________ I am a member of a data collector a research on knowledge of vaccine handlers and status of cold chain and vaccine management in primary health care facilities of Tigray region, Northern Ethiopia which is conducting by Tigray health research institute. This study is commencing in primary health care facilities of 4 selected districts of Tigray region and your health facility is among these selected primary health care facilities. I am one of the data collectors and I am going to ask you some questions related to your socio-demographic and your knowledge on cold chain and vaccine management. Additionally, I will observe and review documents on cold chain and vaccine status and availability of cold chain equipment and tools in your health facilities The objective of this study is to identify the gaps of cold chain and vaccine management. Your name will not be written in this form and will never be used in connection with any of the information you tell me. However, your honest answers to these questions will help to solving the problem on cold chain and vaccine management in your facility as well as in Tigray Region. The interview will take about 45-60 minutes and we are appreciating your help in responding to this research questions. You have a right not to participate and withdraw at any time of interview. Would you be willing to participate? Yes……., No……

**Consent form**

I have been briefly informed about the study and clearly understood the objective of the study. So I am agreeing to participate in this study.

Signature ………………………

**Result**

Result codes: Completed=1, Refused=2, partially completed=3, respondent no found=4

Name of interviewer ………………………..signature………………..date……………….

Interviewer code…………………..

Name of supervisor ………………………….signature………………...date………………

| Annex: II Questionnaire for cold chain and vaccine management | | | | | |
| --- | --- | --- | --- | --- | --- |
| S.N | | Question | Response | Skip | Remark |
| **Part I. Socio-demographic characteristics** | | | | | |
| 101 | | Name of district | 1= T/Machew  2= Werieleke  3= S/Samre  4=R/Alamata |  |  |
| 102 | | Type of health facility? | 1= Health post  2= Health center  3= Primary Hospital  99= Other specify …….. |  |  |
| 103 | | What is your age in complete years? | ……………years |  |  |
| 104 | | Sex of respondent? | 1=Male  2=Female |  |  |
| 105 | | What is your marital status currently? | 1=Single  2=Married  3=Divorced  4=Widowed  5=Separated  99= Other specify……… |  |  |
| 106 | | Profession of respondents? | 1=Health Extension worker  2=Nurse  3=Midwifery  4= Health officer  99= Other specify…. |  |  |
| 107 | | Level of education? | 1=Certificate  2= Diploma  3= Degree  4= Master  99= specify other…….. |  |  |
| 108 | | Total numbers of health workers trained in cold chain management in the health facility? | ……………. |  |  |
| 109 | | Work experience in complete years? | Total Service ………  Related to cold chain … |  |  |
| 110 | | Do you receive training related to cold chin and vaccine management? | 1=Yes  2=No | If 2 Skip to Q201 |  |
| 111 | | If yes, which types of training?  (**Multiple answer is possible** ) | 1= Cold chain management  2= Vaccine management  3= Immunization in Practice  99= Other specify …….. |  |  |
| 112 | | If yes, time of last training? | Before ………. |  |  |
| **Part II. knowledge of vaccine handlers on cold chain and vaccine management** | | | | | |
| 201 | | Do you know how to condition icepacks and how to pack transport boxes? **(Check by demonstration)** | 1=Yes  2=No |  |  |
| 202 | | Do you know how to prevent vaccine freezing during transport? **(Check by demonstration)** | 1=Yes  2=No |  |  |
| 203 | | Do you use freeze indicators during every transport? | 1=Yes  2=No |  |  |
| 204 | | Do you know antigens eligible for the four weeks open vial policy? | 1=Yes  2=No | If 2 Skip to Q206 |  |
| 205 | | If, yes can tell me please?(Lists all during she/he says) | ………………. |  |  |
| 206 | | Are opened multi-dose vial policy (MDVP) kept for the next immunization sessions with visible labeled? **(Check by observation)** | 1=Yes  2=No | If 2 Skip to Q208 |  |
| 207 | | Does opened multi-dose vial will be used for the next session, the vials must be placed in a separate container in the refrigerator, which is clearly marked “Opened vials – Use first.”?  **(Check by observation )** | 1=Yes  2=No  3=Not available |  |  |
| 208 | | Do you know after how many hours the reconstituted vaccines are discarded? | 1=Yes  2=No | If 2 Skip to Q210 |  |
| 209 | | If yes after how many hours? | After……….hrs. |  |  |
| 210 | | Do you know how many hours fridge should be turned on after its arrival? | ………………… | If 2 Skip to Q212 |  |
| 211 | | If yes hours after arrival? | ………………… |  |  |
| 212 | | Do you know at what temperature range should vaccines stored in refrigerator? | 1=Yes  2=No | If 2 Skip to Q214 |  |
| 213 | | If yes at what temperature range? | ………………… |  |  |
| 214 | | Do you know for how many month/s vaccines should be store in the HF? | 1=Yes  2=No | If 2 Skip to Q216 |  |
| 215 | | If yes for how many months? | ………………… |  |  |
| 216 | | Do you know the duration of vaccine stored in the vaccine carrier? | 1=Yes  2=No | If 2 Skip to Q219 |  |
| 217 | | If yes for how many hours? | …………. |  |  |
| 218 | | If yes, in what condition? | ……………………….. |  |  |
| 219 | | Do you know the duration of vaccine store in the cold box? | 1=Yes  2=No | If 2 Skip to Q222 |  |
| 220 | | If yes for how many days? | …………. |  |  |
| 221 | | If yes, in what condition? | ……………………… |  |  |
| 222 | | Could you name all heat sensitive vaccines? | ………. |  |  |
| 223 | | Could you name all cold sensitive vaccines? | ………. |  |  |
| 224 | | Could you name all light sensitive vaccines? |  |  |  |
| 225 | | Correct demonstration of temperature reading? | 1=Yes  2=No |  |  |
| 226 | | Health worker know how to  Read and interpret VVM? **(demonstration)** | 1=Yes  2=No |  |  |
| 227 | | Health worker know when to override EEFO based on VVM status? **(demonstration)** | 1=Yes  2=No |  |  |
| 228 | | Place vaccines with VVMs that show the most heat exposure (darker squares) in a separate container in the refrigerator, clearly marked “Heat-exposed vials – Use first”.  **(Check by observation)** | 1=Yes  2=No  3= Not applicable |  |  |
| 229 | | Does the HF have made following Early Expiry First Out (EEFO) in practice? | 1=Yes  2=No |  |  |
| 230 | | Old and new vaccines organized to facilitate use of older vaccines first  (always)?**(observation the arrangement)** | 1=Yes  2=No |  |  |
| 231 | | The health personnel know the correct interpretation of shake test? **(demonstration)** | 1=Yes  2=No |  |  |
| 232 | | Correct placing thermometer inside deep freezer? **(demonstration)** | 1=Yes  2=No |  |  |
| 233 | | Do you use a foam pad at the top of the vaccine carrier during immunization sessions? | 1=Yes  2=No | If 2 Skip to Q235 |  |
| 234 | | If yes how often | 1= Always  2= Sometimes  3= Rarely  99= Other specify ……. |  |  |
| 235 | | Health workers always use  diluents and vaccine from same  manufacturer and with  Matching presentations?  (**Check by observation**) | 1=Yes  2=No |  |  |
| 236 | | If the diluents are placed at outside refrigerator, does the diluents stored at the correct temperature (cooled to 2–8°C) during immunization sessions before used? | 1=Yes  2=No | If 2 Skip to Q238 |  |
| 237 | | If yes before how many hours? | …………… |  |  |
| 238 | | Health workers know how to calculate the wastage rate**?(demonstration)** | 1=Yes  2=No |  |  |
| **Part III. Cold chain status in the health facility** | | | | | |
| 301 | Available person responsible for cold chain management? | | 1=Yes  2=No |  |  |
| 302 | Cold chain room separated? (**Check by observation**) | | 1=Yes  2=No |  |  |
| 303 | Does the room is clean and ventilated?  (**Check by observation**) | | 1=Yes  2=No |  |  |
| 304 | Facility has adequate cold chain storage space?  (**Check by observation**) | | 1=Yes  2=No |  |  |
| 305 | Availability of at least one functional refrigerator in health facilities? (**Check by observation**) | | 1=Yes  2=No |  |  |
| 306 | Does the refrigerator placed over wooden platform?  (**Check by observation**) | | 1=Yes  2=No |  |  |
| 307 | Does the refrigerator 10 cm away from wall?(**Check by observation**) | | 1=Yes  2=No |  |  |
| 308 | Type of power supply for fridge? | | 1=Electricity as main source  2= solar as main source  3=Kerosene as main source  99=Other specify …… |  |  |
| 309 | During the past six months, was sufficient fuel available to allow all deliveries to be completed on time? (**For refrigerators (if used kerosene and vehicles)?** | | 1=Yes  2=No  3= Not applicable |  |  |
| 310 | Availability of functional Temperature monitoring device in refrigerator?  (**Check by observation**) | | 1=Yes  2=No | If 2 Skip to Q312 |  |
| 311 | If yes, which type?  (**Check by observation**) | | 1=Thermometer  2= Fridge-Tag |  |  |
| 312 | Do you daily temperature recorded? (**Check by observation**) | | 1=Yes  2=No | If 2 Skip to Q319 |  |
| 313 | Does the temperature recorded properly (twice daily and complete) in the past six months?  (**Check by observation**) | | 1=Yes  2=No |  |  |
| 314 | If recording temperature skipped how many days in six months?**(Check by observation)** | | ……………. |  |  |
| 315 | Refrigerators with temperature within the recommended range (2-8°C) at the moment data collection? **(Check by observation)** | | 1=Yes  2=No |  |  |
| 316 | Numbers of temperature out of the +2 to +8 during the six previous months to data collection?  **(Check by observation)** | | …………….. low alarms  ……………high alarms |  |  |
| 317 | Do you formal review of temperature records and excursions and remedial actions?  **(see the document)** | | 1=Yes  2=No | If 2 Skip to Q319 |  |
| 318 | If yes, period of formal review of temperature records?  **(See the plan and document)** | | 1=At least once a month  2= 1-2 months  3= Every 3 month  4= irregularly  99= specify other……… |  |  |
| 319 | Proper storage of vaccines in the refrigerator?  **(Check by observation)** | | 1=Yes  2=No |  |  |
| 320 | Is there any vaccine which reaches the VVM discarding stage in fridge during the visit?  **(Check by observation)** | | 1=Yes  2=No |  |  |
| 321 | Is there any vaccine which reaches expired date in fridge during the visit? **(Check by observation)** | | 1=Yes  2=No |  |  |
| 322 | Proper parking of ice packs in ice-pack freezing compartment?  **(Check by observation)** | | 1=Yes  2=No |  |  |
| 323 | How many water-packs in the freezer/cooling compartment at the moment data collection?  **(Check by observation)** | | ……………………. |  |  |
| 324 | Proper parking of diluents in refrigerators?  **(Check by observation)** | | 1=Yes  2=No |  |  |
| 325 | Deep freezer frost < 5 cm?  **(Check by observation)** | | 1=Yes  2=No |  |  |
| 326 | Does the refrigerator clean? **(Check by observation)** | | 1=Yes  2=No |  |  |
| 327 | Refrigerator used exclusively for vaccine storage (free from other products than vaccine, diluents and ice packs? **(Check by observation)** | | 1=Yes  2=No |  |  |
| 328 | If not what kinds of products stored other than vaccine during visit?  **(Check by observation)** | | ………………………… |  |  |
| 329 | For how many months does the health facility store vaccines (averagely)? | | ……..months |  |  |
| 330 | Does the health facility run out of stock for vaccines in the last six months? | | 1=Yes  2=No | If 2 Skip to Q332 |  |
| 331 | If yes which vaccines | | ……………………… |  |  |
| 332 | Do you have discarded vaccines due to incorrect storage temperatures in the last six months? | | 1=Yes  2=No | If 2 Skip to Q334 |  |
| 333 | If yes please specifies all discarded vaccines due to incorrect storage temperatures in the last six months? | | ………………………… |  |  |
| 334 | Do you recorded all vaccine discarded due to incorrect storage temperatures?  **(Check by observation)** | | 1=Yes  2=No |  |  |
| 335 | Do you have safely disposed of damaged or expired vaccine? | | 1=Yes  2=No |  |  |
| 336 | Available person responsible for cold chain equipments preventive maintenance? | | 1=Yes  2=No |  |  |
| 337 | Do you plan preventive maintenance to cold chain equipment?  **(Check by observation)** | | 1=Yes  2=No |  |  |
| 338 | Did you conduct preventive maintenance to cold chain equipment in the past one year?( see maintenance agreement and service records)  **(Check by observation)** | | 1=Yes  2=No |  |  |
| 339 | Does the HF conduct cold chain equipment inventory?  **(see document)** | | 1=Yes  2=No |  |  |
| **Part IV. Vaccine management/ stock management** | | | | | |
| 401 | Do you have a  Vaccination micro plan?  **(Check by observation)** | | 1=Yes  2=No |  |  |
| 402 | Are standard vaccine requisition forms used for ordering and receiving vaccine?**(Check by observation)** | | 1=Yes  2=No |  |  |
| 403 | Does health facility have recorded minimum - maximum stock levels? **(Check by observation)** | | 1=Yes  2=No |  |  |
| 404 | Physical stock checks are completed each time a monthly or before ordering the next request? | | 1=Yes  2=No |  |  |
| 405 | Did the health facility experienced shortage/ under stock of any vaccines within the last six months? | | 1= Yes  2= No | If 2 Skip to Q408 |  |
| 406 | If yes which vaccines? | | ………………… |  |  |
| 407 | If yes what is the reason? | | …………………….. |  |  |
| 408 | Did the health facility experienced over-stocking of any vaccines within the last six months? | | 1=Yes  2=No | If 2 Skip to Q411 |  |
| 409 | If yes which vaccines? | | ………………… |  |  |
| 410 | If yes what is the reason? | | ………………….. |  |  |
| 411 | Do you have formally communicated vaccine distribution system is in place? (to all respective health facilities) | | 1=Yes  2=No  3=Not applicable |  |  |
| 412 | Does the vaccine distribution plan is implemented in timely fashion? **(Check by observation)** | | 1=Yes  2=No |  |  |
| 413 | Does the health facility have vaccine contingency plan to protect the vaccines in case of any emergency (25-50%)?  **(Check by observation)** | | 1=Yes  2=No |  |  |
| 414 | Does the health facility have sufficiency of dry storage – for syringes & diluents?  **(Check by observation)** | | 1=Yes  2=No |  |  |
| 415 | Are vaccine and diluents quantities (in doses) recorded?  **(Check by observation)** | | 1=Yes  2=No |  |  |
| 416 | Are vaccine and diluents type recorded?**(Check by observation)** | | 1=Yes  2=No |  |  |
| 417 | Are vaccine and diluents manufacturer recorded?  **(Check by observation)** | | 1=Yes  2=No |  |  |
| 418 | Are vaccine and diluents batch/lot numbers recorded?  **(Check by observation)** | | 1=Yes  2=No |  |  |
| 419 | Are vaccine and diluents expiry dates recorded?  **(Check by observation)** | | 1=Yes  2=No |  |  |
| 420 | Are AD syringes amount recorded?**(Check by observation)** | | 1=Yes  2=No |  |  |
| 421 | Is VVM status recorded?  **(Check by observation)** | | 1=Yes  2=No |  |  |
| 422 | Do you calculate vaccine wastage rate? | | 1=Yes  2=No | If 2 Skip to Q501 |  |
| 423 | If yes, the total wastage rate in the past 6 months for each vaccine? **(Check by observation)** | | DPT-HepB-Hib vaccine…  PCV……………  Rota vaccine……..  IPV/ OPV……………  BCG………….  Measles …………..  TT…………  Other………. |  |  |
| 424 | Does wastage rate for all vaccine is in the standard range in the past six months?**( Observation**) | | 1=Yes  2=No |  |  |
| **Part V: Supportive supervision related questions** | | | | | |
| 501 | | Did you have been supervised for cold chain at least the last 6 months? | 1=Yes  2=No | If 2 Skip to Q601 |  |
| 502 | | If yes, frequency of supportive supervisor | …….. |  |  |
| 503 | | From whom | 1= internal staff  2= From catchment health center  3= From district health office  4=From TRHB  5= From partner’s staff  99= other specify…. |  |  |
| 504 | | Availability of feed-back of supportive supervision?  (**see the copy)** | 1=Yes  2=No |  |  |
| **Part VI: Availability of vaccine equipments and recording and reporting tools ( check all by observation)** | | | | | |
| 601 | | Numbers of refrigerators available in HF? **( Observation**) | Functional …………..  Dysfunctional ………. |  |  |
| 602 | | Numbers of ice parks available in HF? **( Observation**) | …….. |  |  |
| 603 | | Numbers of vaccine carrier available in HF? **( Observation**) | …….. |  |  |
|  | | Does health facility have enough AD syringes? **( Observation**) | 1=Yes  2=No |  |  |
| 604 | | Availability of transportation (vehicle or motorcycle)? | 1=Yes  2=No |  |  |
| 605 | | Availability of standard vaccine requisition forms?  **( Observation**) | 1=Yes  2=No |  |  |
| 606 | | Availability of vaccine stock register? **( Observation**) | 1=Yes  2=No |  |  |
| 6907 | | Presence of the national guidelines on immunization? **( Observation**) | 1=Yes  2=No |  |  |
| 608 | | Are written instructions on the use of VVMs, such as posters and stickers, available to storekeepers and health workers?  **( Observation**) | 1=Yes  2=No |  |  |
| 609 | | Cold chain equipment inventory checklist? **( Observation**) | 1=Yes  2=No |  |  |
| 610 | | Availability of temperature recording sheets?  **( Observation**) | 1=Yes  2=No |  |  |
| 611 | | Does your health facility have incineration?  **( Observation**) | 1=Yes  2=No |  |  |
| 612 | | Does your health facility have enough safety boxes?  **( Observation**) | 1=Yes  2=No |  |  |

**Thank you very much!!**
