# Supplementary material for: Knowledge of vaccine handlers and status of cold chain and vaccine management in primary health care facilities of Tigray region, Northern Ethiopia: Institutional based cross-sectional study
Source: PLoS One. 2022 Jun 1;17(6):e0269183. doi: 10.1371/journal.pone.0269183 (PMC9159613; doi:10.1371/journal.pone.0269183)
Supplement: S1 Table — (DOCX) [file pone.0269183.s003.docx]

| **Name of district** | **Numbers of primary health care facilities with functional vaccine refrigerator** | | | | **Numbers of primary health care facilities with no functional vaccine refrigerator** | | | |
| --- | --- | --- | --- | --- | --- | --- | --- | --- |
|  | **Health posts** | **Health centers** | **Primary hospitals** | **Total** | **Health posts** | **Health centers** | **Primary hospitals** | **Total** |
| **Tahtay-Maychew** | 3 | 3 | 1 | 7 | 11 | 0 | 0 | 11 |
| **Werie-Leke** | 16 | 5 | 1 | 22 | 12 | 0 | 0 | 12 |
| **Saharti-Samre** | 5 | 6 | 1 | 12 | 12 | 0 | 0 | 12 |
| **Raya-Alamata** | 7 | 5 | NA | 12 | 7 | 0 | NA | 7 |
| **Total** | 31 | 19 | 3 | 53 | 42 | 0 | 0 | 42 |

**Table . Availability of functional vaccine refrigerators at primary health care facilities in selected districts of Tigray region, Northern Ethiopia 2019**

NA= not applicable
