# Supplementary material for: Knowledge of vaccine handlers and status of cold chain and vaccine management in primary health care facilities of Tigray region, Northern Ethiopia: Institutional based cross-sectional study
Source: PLoS One. 2022 Jun 1;17(6):e0269183. doi: 10.1371/journal.pone.0269183 (PMC9159613; doi:10.1371/journal.pone.0269183)
Supplement: S2 Table — (DOCX) [file pone.0269183.s004.docx]

| **Variables** | **Value** |
| --- | --- |
| Know vaccines stored at a temperature between +2°C to +8°C in the PHCF | 1=Yes, 0=No |
| Know diluents stored at correct temperature (cooled to 2–8°C) during immunization sessions at least 24 hours before use | 1=Yes, 0=No |
| Correctly demonstrate how to pack ice pack to vaccine carrier during transport | 1=Yes, 0=No |
| Know how to prevent vaccine freezing during transport | 1=Yes, 0=No |
| Name all most heat sensitive vaccines (OPV, Measles and BCG) | 1=Yes, 0=No |
| Name all cold sensitive vaccines ( (DPT-HepB-Hib, PCV,TT, IPV and Rota ) | 1=Yes, 0=No |
| Name all light sensitive vaccines (BCG and measles) | 1=Yes, 0=No |
| Correctly read and interpretation of VVM stage | 1=Yes, 0=No |
| Correctly demonstrate and interpretation of shake test | 1=Yes, 0=No |
| Name all vaccines eligible for the four weeks open vial policy(TT,IPV and OPV) | 1=Yes, 0=No |
| Know Early Expiry First Out (EEFO based on VVM status | 1=Yes, 0=No |
| Know Early Expiry First Out (EEFO) based on expiry date | 1=Yes, 0=No |
| Know how to organize old and new vaccines to facilitate use of older vaccines first | 1=Yes, 0=No |
| Know vaccines should be stored only for a maximum of 1 month in PHCF | 1=Yes, 0=No |
| Know diluents and vaccine should be from same manufacturer | 1=Yes, 0=No |
| Know how to calculate the wastage rate | 1=Yes, 0=No |

**Table . Variables computed for the Knowledge of vaccine handlers**
