# Supplementary material for: Knowledge of vaccine handlers and status of cold chain and vaccine management in primary health care facilities of Tigray region, Northern Ethiopia: Institutional based cross-sectional study
Source: PLoS One. 2022 Jun 1;17(6):e0269183. doi: 10.1371/journal.pone.0269183 (PMC9159613; doi:10.1371/journal.pone.0269183)
Supplement: S3 Table — (DOCX) [file pone.0269183.s005.docx]

| **Indicators** | **Value** |
| --- | --- |
| Proper arrangement of vaccines in the refrigerators | 1=Yes, 0=No |
| Proper packing of diluents in the refrigerators | 1=Yes, 0=No |
| Proper packing of ice packs in the refrigerators | 1=Yes, 0=No |
| Refrigerators without expired vaccines date during the visit | 1=Yes, 0=No |
| Refrigerators without VVM discarding stage vaccines during the visit | 1=Yes, 0=No |
| Recording twice daily temperature | 1=Yes, 0=No |
| Using foam pad during immunization sessions (always) | 1=Yes, 0=No |
| Checking physical stock every month before ordering the next request vaccines | 1=Yes, 0=No |
| Using standard vaccine requisition format for ordering and receiving vaccines | 1=Yes, 0=No |
| Properly registered all vaccines in the stock register | 1=Yes, 0=No |
| Did not experience under- stock of any vaccine within the last six months | 1=Yes, 0=No |
| Did not experience over-stock of any vaccine within the last six months | 1=Yes, 0=No |
| Calculating vaccine wastage rate every month within the last six months | 1=Yes, 0=No |

**Table . Variables computed for the cold chain and vaccine management status**
