# Supplementary material for: Knowledge of vaccine handlers and status of cold chain and vaccine management in primary health care facilities of Tigray region, Northern Ethiopia: Institutional based cross-sectional study
Source: PLoS One. 2022 Jun 1;17(6):e0269183. doi: 10.1371/journal.pone.0269183 (PMC9159613; doi:10.1371/journal.pone.0269183)
Supplement: S1 Checklist — (DOCX) [file pone.0269183.s006.docx]

STROBE Statement—Checklist of items that should be included in reports of **cross-sectional studies**

Study Title: **Knowledge of vaccine handlers and status of cold chain and vaccine management in primary health care facilities of Tigray region, Northern Ethiopia: Institutional based cross-sectional study**

|  | Item No | Recommendation |
| --- | --- | --- |
| **Title and abstract** | 1 | (*a*) Indicate the study’s design with a commonly used term in the title or the abstract  In the title page 1 ; line 3  In the abstract: page 2 ; line 34 |
|  |  | (*b*) Provide in the abstract an informative and balanced summary of what was done and what was found Abstract: Page 2, lines from 34-50. |
| Introduction | | |
| Background/rationale | 2 | Explain the scientific background and rationale for the investigation being reported Introduction: page 4-5 ,lines 92-100 |
| Objectives | 3 | State specific objectives, including any prespecified hypotheses Introduction: page 4-5 , and lines 95-100 |
| Methods | | |
| Study design | 4 | Present key elements of study design early in the paper Methods (on study design sub section ) page 5; lines 119-121 |
| Setting | 5 | Describe the setting, locations, and relevant dates, including periods of recruitment, exposure, follow-up, and data collection Methods (study setting and period sub section )page 5; lines 103-110. |
| Participants | 6 | (*a*) Give the eligibility criteria, and the sources and methods of selection of participants Methods (study participant sub section) page 6; line 122-126 |
| Variables | 7 | Clearly define all outcomes, exposures, predictors, potential confounders, and effect modifiers. Give diagnostic criteria, if applicable Methods (measurement of variables sub section) page 7-8; lines 151-178 |
| Data sources/measurement | 8* | For each variable of interest, give sources of data and details of methods of assessment (measurement). Describe comparability of assessment methods if there is more than one group Methods (data collection procedures and data quality assurances sub section) page 6; lines 140-143. |
| Bias | 9 | Describe any efforts to address potential sources of bias Methods (data collection procedures and data quality assurances sub section ) page 6-7; line 144-149. |
| Study size | 10 | Explain how the study size was arrived at Methods (sampling methods and procedure sub section) page 6; lines 128-133. |
| Quantitative variables | 11 | Explain how quantitative variables were handled in the analyses. If applicable, describe which groupings were chosen and why Methods (data management and analysis sub section) page 8; lines 189-196 |
| Statistical methods | 12 | (*a*) Describe all statistical methods, including those used to control for confounding Methods (data management and analysis sub section) page 9; lines 196-199 |
|  |  | (*b*) Describe any methods used to examine subgroups and interactions |
|  |  | (*c*) Explain how missing data were addressed |
|  |  | (*d*) If applicable, describe analytical methods taking account of sampling strategy |
|  |  | (*e*) Describe any sensitivity analyses |
| Results | | |
| Participants | 13* | (a) Report numbers of individuals at each stage of study—eg numbers potentially eligible, examined for eligibility, confirmed eligible, included in the study, completing follow-up, and analysed Results page 9; lines 212-213 |
|  |  | (b) Give reasons for non-participation at each stage |
|  |  | (c) Consider use of a flow diagram |
| Descriptive data | 14* | (a) Give characteristics of study participants (eg demographic, clinical, social) and information on exposures and potential confounders Results page 9; lines 213-216 (table 1) |
|  |  | (b) Indicate number of participants with missing data for each variable of interest |
| Outcome data | 15* | Report numbers of outcome events or summary measures Results page 11-13 ; lines 222-253 (table 2 and 3) |
| Main results | 16 | (*a*) Give unadjusted estimates and, if applicable, confounder-adjusted estimates and their precision (eg, 95% confidence interval). Make clear which confounders were adjusted for and why they were included Results page 17; lines 264-270 (table 5) and page 18; line 279-288 (table 6). |
|  |  | (*b*) Report category boundaries when continuous variables were categorized Results page 12; lines 229-231and page 13; lines 251-253. |
|  |  | (*c*) If relevant, consider translating estimates of relative risk into absolute risk for a meaningful time period |
| Other analyses | 17 | Report other analyses done—eg analyses of subgroups and interactions, and sensitivity analyses |
| Discussion | | |
| Key results | 18 | Summarise key results with reference to study objectives Discussion page 19-23; lines 295-412 |
| Limitations | 19 | Discuss limitations of the study, taking into account sources of potential bias or imprecision. Discuss both direction and magnitude of any potential bias Discussion (strength and limitation sub section) page 18; lines 388-392 |
| Interpretation | 20 | Give a cautious overall interpretation of results considering objectives, limitations, multiplicity of analyses, results from similar studies, and other relevant evidence Conclusion page 24; lines 417-422 |
| Generalisability | 21 | Discuss the generalisability (external validity) of the study results bias Discussion (strength and limitation sub section) page 24; lines 419-422 |
| Other information | | |
| Funding | 22 | Give the source of funding and the role of the funders for the present study and, if applicable, for the original study on which the present article is based  The study was funded by Tigray regional government. The funder had no role in the study design, data collection and analysis, and preparation of the manuscript. |

*Give information separately for exposed and unexposed groups.

**Note:** An Explanation and Elaboration article discusses each checklist item and gives methodological background and published examples of transparent reporting. The STROBE checklist is best used in conjunction with this article (freely available on the Web sites of PLoS Medicine at http://www.plosmedicine.org/, Annals of Internal Medicine at http://www.annals.org/, and Epidemiology at http://www.epidem.com/). Information on the STROBE Initiative is available at www.strobe-statement.org.
